# Strategies and safety considerations of booster vaccination in COVID-19

**DOI:** 10.17305/bjbms.2021.7082

**Published:** 2022-04-03

**Authors:** Hanyan Meng, Jianhua Mao, Qing Ye

**Affiliations:** The Children’s Hospital, Zhejiang University School of Medicine, National Clinical Research Center for Child Health, National Children’s Regional Medical Center, Hangzhou, China

**Keywords:** SARS-CoV-2, COVID-19 vaccine, Omicron, booster vaccination, heterologous vaccination

## Abstract

The first-generation SARS-CoV-2 vaccines have played a significant role in controlling the COVID-19 pandemic, preventing severe diseases, and reducing mortality. However, the continuous emergence of SARS-CoV-2 variants, the persistence of breakthrough infections, and the seemingly rapid decline in the protective efficacy of SARS-CoV-2 vaccines have presented additional challenges for the next phase. There is an urgent need to confirm the necessity of further booster vaccination and combination vaccine approaches. This paper summarizes the latest literature on SARS-CoV-2 variants and vaccine effectiveness and concludes that it is essential to implement booster immunization strategies. Priority should be given to high-risk groups, the elderly, and immunocompromised people. In addition, heterologous vaccination has a longer duration of effect and a broader spectrum than homologous vaccination, making it more conducive to managing the immune escape of SARS-CoV-2 variants.

## INTRODUCTION

Vaccination has been proven to be the most effective and economical mean to prevent and control the COVID-19 epidemic [1]. Since the outbreak of COVID-19, countries around the world have been actively engaged in the development and inoculation of COVID-19 vaccines [1-3]. As of March 2022, the World Health Organization (WHO) has reported 334 candidates of COVID-19 vaccines that are in trials, among which 149 are in the clinical phase and 195 in the pre-clinical phase [4]. However, while the entire world is actively responding to COVID-19, the virus itself is constantly changing [5,6]. A variety of SARS-CoV-2 variants designated by the WHO have emerged: Alpha (B.1.1.7), Beta (B.1.351), Gamma (P.1), Delta (B.1.617.2), and more recently Omicron (B.1.1.529) [7]. Supervirulent Omicron, first reported in South Africa in November 2021, is spreading rapidly with super infectious power. There appeared to be early signs that the existing vaccines might not be able to withstand Omicron in December 2021, and some studies have confirmed that Omicron can escape immunity [8-10]. Corti et al. [11] demonstrated that Omicron might have a stronger immune escape ability than previous SARS-CoV-2 variants. In addition, the titers of neutralizing antibodies produced by the body after vaccination will gradually decrease over time. Booster vaccination may be needed to further strengthen the body’s immunity against SARS-CoV-2 [12,13].

We performed a literature search of the PubMed database from inception until February 2022 using combinations of the search terms “SARS-CoV-2,” “COVID-19,” “vaccine,” and “booster vaccination.” We also searched reference lists of identified articles and other relevant articles on vaccine effectiveness, booster vaccination, and heterologous vaccination. We excluded studies that were not human studies or only for people with certain diseases. Finally, we summarized and analyzed the necessity of booster vaccination, priority groups for vaccination, heterologous booster vaccination strategies, and safety considerations of booster vaccination. We sincerely hope that our review would better guide booster vaccination strategies for COVID-19 vaccines.

## THE NECESSITY OF BOOSTER VACCINATION

Vaccine-induced immune protection diminishes over time after vaccination, which is the natural law of immune response and the constant topic of vaccine persistence research [14,15]. Whether it is inactivated vaccine, recombinant protein vaccine, adenovirus vector vaccine, or mRNA vaccine, immune regression will gradually occur after approximately six months after the completion of the immunization program [12,16,17]. Tartof et al. [18] showed that the immunoprotective effectiveness of BNT162b (Pfizer-BioNTech) vaccine against infections caused by the Delta variant decreased from 93% at the first month after being fully vaccinated to 53% at the fourth month after full vaccination. The effectiveness against other (non-Delta) variants decreased from 97% within 1 month of being fully vaccinated to 67% up to 5 months after being fully vaccinated. Pegu et al. [19] assessed the neutralizing effect of different SARS-CoV-2 variants that waned after approximately 6 months after second dose with mRNA-1273, and found that 96%, 96%, 88%, 85%, and 54% of sera neutralized the variants B.1.1.7 (Alpha), B.1.617.2 (Delta), B.1.526 (Iota), P.1 (Gamma), and B.1.351 (Beta), respectively. Across all assays, B.1.351 had the lowest antibody recognition. In another related study, we learned the effectiveness of a one-dose regimen of Ad26. COV2.S (Janssen) against COVID-19 was 74.8% at 1 month and decreased to 59.4% at 5 months (the original article did not discuss which variants the listed data target for) [20]. Reassuringly, studies show that mutations in variants of concern (Alpha, Beta, Gamma, and Delta) do not impact the T-cell response elicited by vaccines or natural infection [21-23]. In addition, the variation in viral antigens helps the virus escape the effective immune response or antibody library of the original lineage [24]. As a result, the initial antibody level is insufficient to exert an adequate immunoprotective effect. It has been reported that even individuals who have been vaccinated with COVID-19 vaccines will still be infected with the Omicron variant [25]. Hoffmann et al. [24] found that the ability of Omicron’s S protein to escape antibodies is 44 times higher than that of the Delta variant, which could lead to ineffective therapeutic antibodies and possibly reduce the protective effect of antibodies induced after infection or two doses of COVID-19 vaccine. In addition, the neutralizing antibody efficacy of sera of vaccine recipients of mRNA-1273 (Moderna), BNT162b, and Ad26.COV2.S against the wild-type, Delta, and Omicron variants was tested. The results showed that neutralization of Omicron could not be detected in most vaccines, and that the Omicron variant can escape vaccine-induced neutralization immunity under the current vaccination scheme [26]. SARS-CoV-2 variants, especially Omicron, have strong immune escape ability. Therefore, it is critical to study the neutralizing activity of various vaccines against Omicron variants as soon as possible [10]. As a result of the aforementioned issue, countries worldwide began to work on booster immunization strategies. Noa et al. [27] evaluated the anti-spike IgG antibody titers of people aged 60 and over before and after the third dose of BNT162b2. The results showed that the neutralizing antibody titers increased from 440 AU/mL to 25,468 AU/ml after the booster dose, and no serious adverse events were reported during this period. Gupta et al. [28] demonstrated that, compared with Delta, Omicron’s spike protein has a higher affinity for angiotensin-converting enzyme 2 (ACE2), and its antigenicity has changed significantly, resulting in significant immune escape. The third dose of the mRNA vaccine can resist the immune escape of Omicron to some extent. In addition, it was pointed out that the titer of neutralizing antibody against Omicron in the serum of recovered patients was only 1/60~1/30 of the original one. However, a booster dose of vaccine can clearly increase the titer of neutralizing antibodies [24,29]. In addition, Schwartz et al. [30] found that the active ingredients against Omicron could hardly be detected in the sera collected five months after the second dose of the BNT162b2 or ChAd0x1-S (Vaxzevria) vaccine. However, after the third dose of the BNT162b2 vaccine, the activity of neutralizing antibodies against Omicron increased nearly six times. Therefore, it is necessary to carry out booster vaccination strategies [13]. Booster vaccination keeps the neutralizing antibody at a high level and has a good cross-synthesis effect on mutant strains such as Omicron [13,26].

## THE PRIORITY POPULATION OF BOOSTER VACCINATION

Studies have shown that sex, age, underlying chronic illness, and immunosuppressant treatment are all related to neutralizing antibody levels after vaccination [31-35]. Therefore, the above factors should be considered in booster immunization and vaccine dosage selection. Elderly individuals have weakened immune system and often have various basic diseases, and therefore are generally more susceptible to COVID-19. The risk of hospitalization, severe illness, and death in elderly individuals after SARS-CoV-2 infection are significantly higher than in adults and children [36]. However, according to statistics, COVID-19 vaccination rates among elderly population vary widely in different countries and regions. Furthermore, the neutralizing antibody levels of the elderly after COVID-19 vaccination decrease rapidly over time, which indicates a deficiency in the immune barrier of the group [37-40]. Therefore, some experts suggest that elderly individuals should not only complete the full vaccination as soon as possible but may also need to be revaccinated earlier or with higher doses [31]. In addition, Furer et al. [41] found that treatment with immunosuppressants such as glucocorticoids and rituximab was related to a significant decrease in immunogenicity induced by BNT162b2. Thuluth et al. [42] found that in a large proportion of patients with liver transplantation, chronic liver diseases, or other immunocompromised states, neutralizing antibodies could not be distinctly detected after vaccination with COVID-19 vaccines. Therefore, people with a severely compromised immune system, including those with advanced HIV, leukemia, organ transplantation, or those receiving immunosuppressive drugs, should also be targeted for booster vaccination [42-44]. Furthermore, some high-risk groups have occupational exposure risks, such as frontline medical and epidemic prevention personnel, port staff, urban operation support personnel, and people going abroad. The priority to booster vaccination should be given to the abovementioned key groups to provide timely immunization protection [45]. Children do not appear to be high on the priority list for COVID-19 vaccination because they have consistently been considered to have a lower burden of COVID-19 disease than adults [46]. Meanwhile, a recent study showed that parental vaccination can substantially reduce the risk of infection among unvaccinated children in the household [47]. However, some countries have implemented vaccine programs for teenagers and even younger children due to the increasing number of children infected with the Omicron variant [48]. A study showed that the hospitalization rates among unvaccinated adolescents were tenfold higher than those among fully vaccinated adolescents [49]. In the long-term perspective, the impact of the COVID-19 pandemic on children is unclear, and it may influence the mood and mental status of affected children [50,51]. Therefore, children should be inoculated with vaccines to fight against COVID-19 illness.

Similarly, the CDC recommends those who work or live in high-risk settings, medical facilities, and long-term care settings, those who completed their initial vaccination scheme a minimum of 6 months ago, or those 65 years and older to receive COVID-19 vaccine booster shots to strengthen the immune barrier [52]. Overall, the current evidence on vaccine prioritization is insufficient; we still need considerable effort to optimize vaccination strategies among special populations, and more data on vaccine effectiveness and safety should be obtained to pave the way for the next stage of expanded vaccination [53].

## THE STRATEGY OF HETEROLOGOUS VACCINATION

**FIGURE 1:**
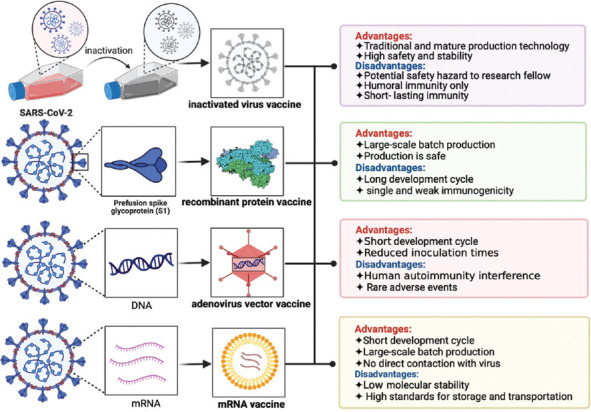
Comparison of four main types of COVID-19 vaccines.

**FIGURE 2:**
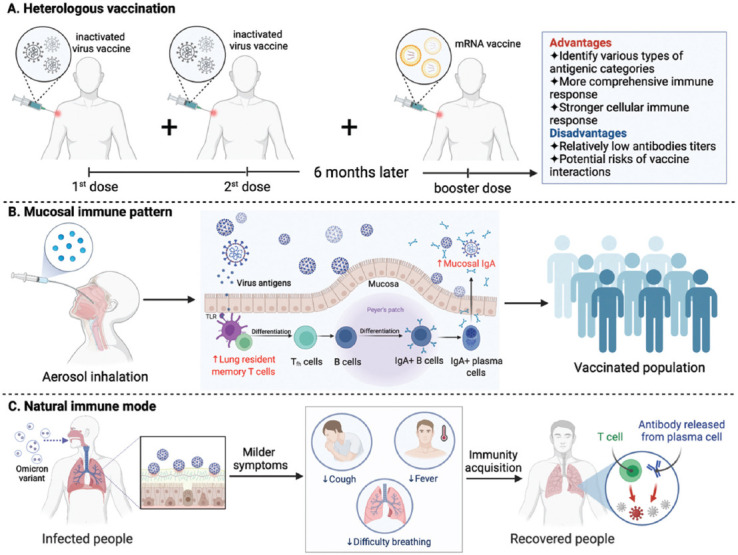
Different immunization methods used in COVID-19. (A) The main process of heterologous vaccination and its advantages and disadvantages; (B) the inhalation delivery of vaccine can cause higher levels of mucosal IgA and lung resident memory T cells; and (C) the Omicron variant can be used as a natural vaccine to help all humans build an immune barrier.

At present, the approved COVID-19 vaccines for the market are mainly divided into the following categories according to the technical route: Adenovirus vector vaccine, recombinant protein vaccine, inactivated virus vaccine, and nucleic acid vaccine (Figure 1). According to the past vaccination experience, the same type of vaccine (or at least the same technical route) is generally used for booster vaccination, i.e., homologous inoculation [54]. However, for COVID-19 vaccination, some scholars have put forward the sequential immunization strategy of heterologous initial and booster immunization, i.e., heterologous vaccination [55]. In booster vaccination, heterologous vaccination refers to using a combination of vaccines from different manufacturers or different technical routes (Figure 2A) [55-59]. Homologous vaccination can trigger a robust immune memory response and rapidly induce a large number of antibodies with higher abundance and maturity, which can maintain a longer duration and a higher protective antibody titer [59,60]. When the strategy of heterologous inoculation is adopted, the immune system will produce a more balanced and comprehensive immune response because the types and amounts of neutralizing antibodies produced by vaccines from different manufacturers vary widely [55,61,62]. Heterologous vaccination can achieve the effect that a single vaccine cannot [63,64]. At present, researchers worldwide are actively exploring the protective effect of heterologous booster immunization [56,57,65,66]. Studies have shown that the mixed vaccination schemes of COVID-19 vaccines can cause higher antibody levels and a more comprehensive immune response, even exceeding the efficacy of the standard vaccination schemes, with no more severe side effects than those caused by the standard schemes [62,65,67]. Other studies have found that people who have been vaccinated twice with inactivated vaccines fail to produce immune molecules that can resist the transmission of Omicron. After the third dose of inactivated vaccine, the level of neutralizing antibodies in individuals remained low. However, it seems to be more effective against Omicron when given a third dose of different vaccine, such as one based on mRNA or recombinant protein [68]. In Chile, approximately two million people who received two doses of the CoronaVac (Beijing, Sinovac) inactivated vaccine were immunized with the CoronaVac, BNT162b2 mRNA vaccine, and ChAd0x1-S adenovirus vector vaccine five months later. The overall protection rate of the vaccine increased from 56% to 80%, 90%, and 93%, respectively [69,70]. A study by Pérez-Then et al. [71] showed that the activity of a specific neutralizing antibody against the Omicron variant could be increased by 1.4 times after using two doses of inactivated CoronaVac vaccine and then injected with BNT162b2. At present, the commonly used COVID-19 vaccine is based on the Wuhan strain of SARS-CoV-2, but that is no longer what the virus itself looks like [72]. Nemet et al. [73] studied the neutralization efficiency against the wild-type SARS-CoV-2 virus and Beta, Delta, and Omicron variant isolates after the third dose of BNT162b2. The neutralization efficiency of the BNT162b2 vaccine against all the tested variants of interest (Beta, Delta, and Omicron) was significantly lower than that against the wild-type virus. Fortunately, mixing different vaccines from different technologies and platforms may extend divergent effectiveness. Therefore, the proposal of heterologous vaccination provides a new idea for COVID-19 vaccination strategies.

Moreover, Roy et al. [74] developed an adenovirus vector vaccine inhaled by aerosol, which has the potential effect of inducing respiratory mucosal immunity. Xu et al. [75] showed that Ad5-nCoV delivered by inhalation robustly elicited both systematic and mucosal immune responses against SARS-nCoV-2 and variants. As a supplementary immunity of systemic immunity, inhalation delivery remains a simple, non-invasive, and cost-effective vaccine administration, which can cause higher levels of mucosal IgA and lung resident memory T cells (Figure 2B) [76,77]. Using a non-injecting vaccine as a booster may become a practical alternative to intramuscular application for the control of COVID-19. Nonetheless, more scientific data and clinical studies are needed to verify which vaccination method can achieve a better immune protection effect [66].

## SAFETY CONSIDERATIONS OF COVID-19 VACCINES

Booster vaccination in COVID-19 has played an essential role in protecting vulnerable groups and alleviating medical and health pressure. However, there are limited data on the safety comparison of COVID-19 vaccines and how they trigger immune responses when administered as a third dose. At the beginning of December 2021, the Lancet published the first randomized clinical trial of booster injection after two doses of the COVID-19 vaccine. The results showed that six different COVID-19 booster shots are safe and have acceptable levels of inflammatory side effects, including pain at the injection site, muscle soreness, fatigue, and headaches [78]. However, there are also reports that some individuals may have serious adverse reactions, such as myocarditis, thrombotic diseases, and Guillain–Barre syndrome [79-81]. Khajavirad et al. [82] reported three cases of serious adverse events after vaccination with ChAd0x1-S, including encephalopathy, vaccine-induced thrombotic thrombopenia, and destructive leukocyte vasculitis. Plüß et al. [83] reported a case of cytomegalovirus reactivation and pericarditis after vaccination with ChAd0x1-S. Nonetheless, information from different clinical trials suggests that vaccine-related side effects of the third doses of vaccines were similar to those observed after the first and second doses of vaccines [84-86].

While serious adverse events after vaccination in COVID-19 are rare, ongoing monitoring of its safety is necessary. Recently, some scholars have proposed an alternative scenario in which the highly mutated but benign Omicron strain could lead us into a relationship of endemicity with a more common cold-like pathogen, meaning that the Omicron variant can be used as a natural vaccine to help all humans build an immune barrier (Figure 2C) [87-89]. Whether the immunity acquired by innate immunity can avoid the risks caused by vaccination and whether there are potential safety hazards in natural immunization itself remain to be confirmed by further research.

## CONCLUSION

Vaccination in COVID-19 to build a herd immunity barrier is the most economical, convenient, and effective measure to prevent and control the COVID-19 pandemic. However, the titers of neutralizing antibodies produced by the body after vaccination gradually decrease over time. Immunization escape may also occur in newly emerging mutant strains, and breakthrough infection may still occur even if the full immunization has been completed. Booster and periodic vaccination will be required for special and compromised populations until a long-lasting vaccine is developed.
